# Insights into the Host Specificity of a New Oomycete Root Pathogen, *Pythium brassicum* P1: Whole Genome Sequencing and Comparative Analysis Reveals Contracted Regulation of Metabolism, Protein Families, and Distinct Pathogenicity Repertoire

**DOI:** 10.3390/ijms22169002

**Published:** 2021-08-20

**Authors:** Mojtaba Mohammadi, Eric A. Smith, Michael E. Stanghellini, Rakesh Kaundal

**Affiliations:** 1 Department of Microbiology and Plant Pathology, University of California, 900 University Ave., Riverside, CA 92521, USA; mojtaba.mohammadi@usda.gov (M.M.); eas10@iu.edu (E.A.S.); mstang@ucr.edu (M.E.S.); 2 Department of Plants, Soils, and Climate, College of Agriculture and Applied Sciences, Utah State University, 4820 Old Main Hill, Logan, UT 84322, USA; 3 Bioinformatics Facility, Center for Integrated BioSystems, College of Agriculture and Applied Sciences, Utah State University, 4700 Old Main Hill, Logan, UT 84322, USA

**Keywords:** *Pythium brassicum*, *Brassicaceae*, whole genome sequence, host-specific mechanisms, root pathogen, comparative genomics, oomycete

## Abstract

*Pythium brassicum* P1 Stanghellini, Mohammadi, Förster, and Adaskaveg is an oomycete root pathogen that has recently been characterized. It only attacks plant species belonging to *Brassicaceae* family, causing root necrosis, stunting, and yield loss. Since *P. brassicum* P1 is limited in its host range, this prompted us to sequence its whole genome and compare it to those of broad host range *Pythium* spp. such as *P. aphanidermatum* and *P. ultimum* var. *ultimum*. A genomic DNA library was constructed with a total of 374 million reads. The sequencing data were assembled using SOAPdenovo2, yielding a total genome size of 50.3 Mb contained in 5434 scaffolds, N50 of 30.2 Kb, 61.2% G+C content, and 13,232 putative protein-coding genes. *Pythium brassicum* P1 had 175 species-specific gene families, which is slightly below the normal average. Like *P. ultimum*, *P. brassicum* P1 genome did not encode any classical RxLR effectors or cutinases, suggesting a significant difference in virulence mechanisms compared to other oomycetes. *Pythium brassicum* P1 had a much smaller proportions of the YxSL sequence motif in both secreted and non-secreted proteins, relative to other *Pythium* species. Similarly, *P. brassicum* P1 had the fewest *Crinkler* (CRN) effectors of all the *Pythium* species. There were 633 proteins predicted to be secreted in the *P. brassicum* P1 genome, which is, again, slightly below average among *Pythium* genomes. *Pythium brassicum* P1 had only one cadherin gene with calcium ion-binding LDRE and DxND motifs, compared to *Pythium ultimum* having four copies. *Pythium brassicum* P1 had a reduced number of proteins falling under carbohydrate binding module and hydrolytic enzymes. *Pythium brassicum* P1 had a reduced complement of cellulase and pectinase genes in contrast to *P. ultimum* and was deficient in xylan degrading enzymes. The contraction in ABC transporter families in *P. brassicum* P1 is suggested to be the result of a lack of diversity in nutrient uptake and therefore host range.

## 1. Introduction

*Pythium* spp. belong to oomycetes, a diverse group of fungal-like organisms that are members of the non-photosynthetic *Staminipila* and closely related to aquatic organisms such as brown algae and diatoms [1]. Within the genus *Pythium*, there are as many as 355 described species [2], of which 116 species and varieties are classified into 11 phylogenetic clades, designated as Clades A–K, based on internal transcribed spacer (ITS) region of the nuclear ribosomal DNA [3]. The majority of *Pythium* species are ubiquitous, soilborne, saprophytic, or facultative necrotrophic root pathogens, causing a wide range of diseases such as stem rots and damping-off, root, stem, and fruit rots, leaf blights, and postharvest decay [4]. They are considered preclinical opportunistic necrotrophs that attack crop species at the seedling stage or under stress [5].

*Pythium* species are genetically diverse and significantly distinct with respect to host range, virulence, and geographical distribution [4,5,6,7]. For instance, *Pythium aphanidermatum* has a broad host range, is extremely virulent, and is a high temperature root pathogen that occurs routinely under greenhouse conditions, whereas *Pythium arrhenomanes* is more restricted to monocots. On the other hand, both *Pythium ultimum* var. *ultimum* and *Pythium irregulare* are highly virulent at cooler temperatures, with broad host range and genetic and morphological diversities. *Pythium iwayamai* is another cool temperature species and causes snow rot on economically important monocots such as barley and winter wheat [8]. *Pythium* species that belong to clade K are phylogenetically distinct from the rest of *Pythium* spp. and are reported to exhibit characteristics shared by both *Pythium* and *Phytophthora*, and are therefore named *Phytopythium* [9]. One such example is *Phytopythium vexans*, which causes root rot in many tropical plants, including durian [10] and rubber tree [11].

To this date, as many as eleven species of *Pythium* have been sequenced using next-generation sequencing platforms. These include *P. aphanidermatum*, *P. arrhenomanes*, *P*. *guiyangense, P. insidiosum, P*. *irregulare*, *P. iwayamai*, *P. oligandrum*, *P. periplocum*, *P*. *splendens*, *P. ultimum* var. *sporangiiferum*, and *P. ultimum* var. *ultimum* [12,13,14,15]. Recent comparative genome analyses have revealed a significant reduction in genome size (genes involved in infection process) in *P. ultimum* var. *ultimum* compared to *Phytophthora* species [12,15].

*Pythium brassicum* Stanghellini, Mohammadi, Förster, and Adaskaveg is an oomycete root pathogen that has recently been characterized based on morphology, host range, and molecular phylogeny [16]. Unlike other *Pythium* spp., *P. brassicum* P1 has a narrow host range and only attacks roots of vegetable crops belonging to the *Brassicaceae* family, thereby causing root rot, stunting, and yield loss.

The main objectives in this study were to investigate how genetically different *P. brassicum* P1 is from broad host range *Pythium* spp. and to understand its pathogenicity mechanisms based on the complete genome sequence analysis and comparative genomics.

## 2. Results and Discussion

### 2.1. Genome Sequencing, Assembly, and Annotation

**Figure 1:**
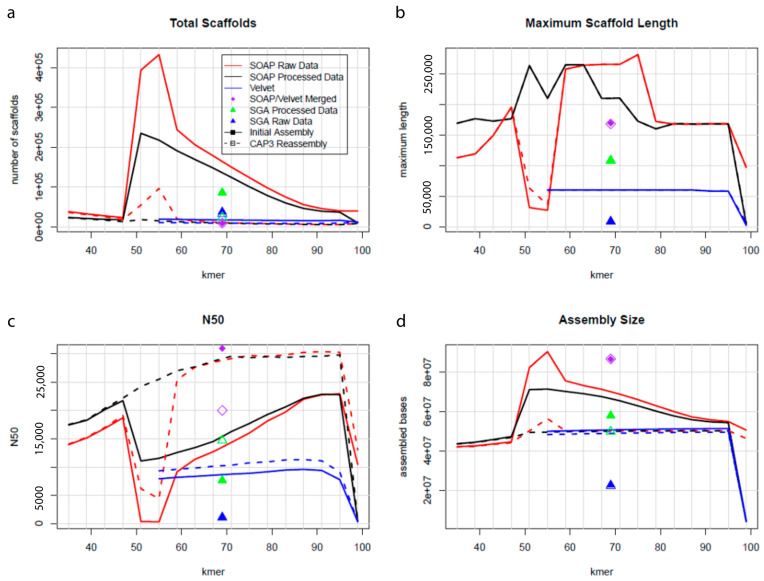
Plots representing total scaffolds (**a**), maximum scaffold length (**b**), N50 statistics (**c**), and assembly size (**d**) of *P. brassicum* P1 genome. The quality of the completed assembled genome was performed using QUAST.

Sequencing was carried out on the Illumina HiSeq 2500, generating a total of 374 million, 1 × 100 bp reads, resulting in 37.4 gigabases of sequence data; 91.4% of bases had a quality score ≥ Q30. An initial SOAPdenovo2 [17] assembly was generated and re-assembled using CAP3 [18]. This assembly was 50.3 Mb, spread among 5434 scaffolds, with a N50 scaffold length of 30.2 Kb, a N90 scaffold length of 6892 bp, and GC content of 61.2% (Table 1 and Table 2). The *P. brassicum* P1 genome was larger and had a higher GC content than *P. ultimum* (genome size: 42.8 Mb, 52.3% G+C content) [19]. The quality of the complete assembled genome was examined using QUAST (Figure 1). The MAKER annotation pipeline [20] predicted 13,232 genes in the *P. brassicum* P1 genome, which fell within the range of previously published *Pythium* genomes. The completeness of the *P. brassicum* P1 was evaluated using BUSCO [21]; *P. brassicum* P1 was missing 27 of 429 (~6%) eukaryotic universal single-copy orthologs, which was again within the range of previously published *Pythium* genomes (genomes downloaded from the *Pythium* Genome Database, and BUSCO scores were determined as with *P. brassicum* P1). RepeatScout [22] identified 49,717 unclassified repeat sequences in the genome, representing 23.35% of the total genomic sequence. Both the total number of repeats and the percentage of the genome contained in repeat sequence were much higher in *P. brassicum* P1 than *P. ultimum*, but considerably lower than *Phytophthora infestans* [23]; interestingly, genome size and GC content in *P. brassicum* P1 also represented an intermediate between *P. ultimum* and *Ph. infestans*.

### 2.2. Annotation of Predicted Proteins

For annotation, the *P. brassicum* P1 assembled sequences were searched against the NCBI non-redundant protein database (NR) with a cut-off E-value of 1 × 10^−6^. There were >30,000 BLAST hits that met this E-value cut-off threshold, indicating that, on average, a predicted gene had ~3 BLAST hits; this provides a robust basis for Gene Ontology (GO) term prediction (see Section 2.3). The most abundant species hit was *Phytophthora parasitica,* another oomycete plant pathogen (Supplementary Figure S1). The majority of hits had 60% positive matches over the length of the alignment.

### 2.3. Classification of Gene Ontology (GO)

**Figure 2:**
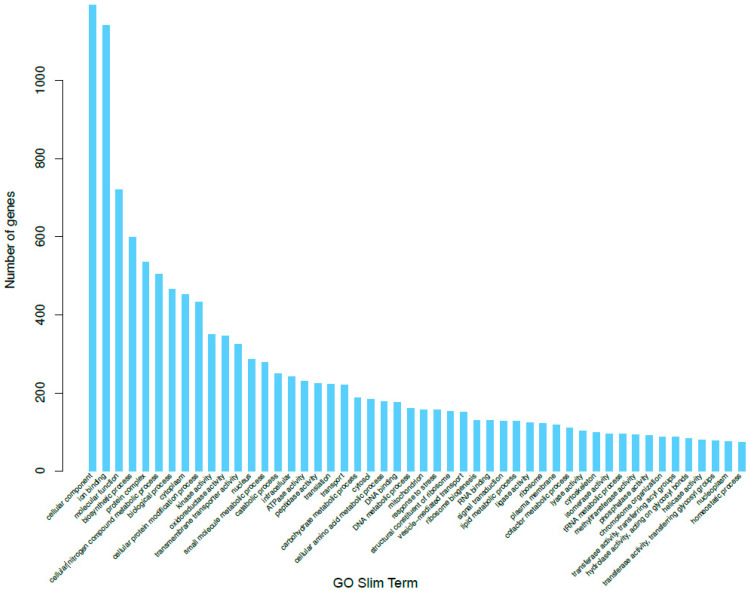
Gene Ontology (GO) distributions for *P. brassicum* P1 predicted genes for all 3 GO categories; Biological Process (BP), Molecular Function (MF), and Cellular Component (CC).

We used the program Blast2GO [24] to convert our BLAST results into GO term annotations. In total, there were 3746 genes annotated in the biological process category, 3033 in the cellular component category, and 3895 in the molecular function category (Figure 2). In the biological process category (Supplementary Figures S2 and S3), the most prominent level 3 GO annotations were cellular metabolic process, organic substance metabolic process, primary metabolic process, single-organism cellular process, and nitrogen compound metabolic process. These processes could all, ostensibly, serve important roles in the pathogenicity of *P. brassicum*, particularly organic substance metabolic processes and nitrogen compound metabolic processes, as these are important components of plant health. In the cellular component category (Supplementary Figures S4 and S5), the most prominent level 3 annotations were intracellular, intracellular part, intracellular organelle, membrane-bound organelle, and intrinsic component of membrane. Intracellular and membrane components may play roles in how *P. brassicum* interacts with its plant hosts, and these genes provide interesting avenues for additional study. In the molecular function category (Supplementary Figures S6 and S7), the most abundant level 3 annotations were heterocyclic compound binding, organic cyclic compound binding, ion binding, hydrolase activity, and transferase activity. Ion binding functions and hydrolase functions have the potential to contribute to plant pathogenicity in *P. brassicum*, as ions are important intracellular signals and could be used by *P. brassicum* as a means of interfering with normal plant biology; hydrolases may be used by *P. brassicum* to break cell wall bonds and infiltrate plant cells.

### 2.4. Over- and Under-Represented Gene Families

We used two methods to determine which gene families were over- or under-represented in the *P. brassicum* P1 genome relative to closely related species. The first method was a comparison of the genome content *P. brassicum* P1 and *P. ultimum* var. *ultimum* using the PANTHER database (pantherdb.org). This analysis uses a Fisher’s exact test with false discovery rate correction to determine significantly over- and under-represented PANTHER families in one genome relative to another. Interestingly, when we compared *P. brassicum* P1 to *P. ultimum* var. *ultimum*, there were no PANTHER families that were significantly over- or under-represented in either genome relative to the other. There were, however, several PANTHER GO-slim biological process families that were more than two-fold enriched in *P. brassicum* P1 relative to *P. ultimum* var. *ultimum*. These included: system process (3.07-fold enriched), neurological system process (3.07-fold enriched), cell growth (2.04-fold enriched), spermatogenesis (2.04-fold enriched), growth (2.04-fold enriched), gamete generation (2.04-fold enriched), and negative regulation of apoptotic process (2.04-fold enriched). It is interesting that the majority of enriched biological process categories in *P. brassicum* are ostensibly involved in cell growth and reproduction. These could be adaptations to increase spread throughout the host plant and between host plant specimens. As a specialist pathogen, it is feasible that *P. brassicum* has adapted to specialize in how it utilizes the nutrients available to it, and thus is able to reproduce and grow faster than closely-related generalist pathogens. In the PANTHER GO-slim molecular function category, there were two families that were greater than two-fold enriched in *P. brassicum* P1: amino acid kinase activity and DNA-methyltransferase activity. Both of these categories may play roles in how *P. brassicum* communicates or interferes with communication of the host plant. There was only one PANTHER family that was greater than two-fold enriched in *P. ultimum* var. *ultimum* relative to *P. brassicum* P1 (e.g., under-represented in *P. brassicum* P1): ectoderm development. The second approach we took to determine over- and under-represented gene families in *P. brassicum* P1 was CAFE, which uses a stochastic birth–death model across a phylogeny to determine which gene families are significantly expanding or contracting (relative to the ancestral state) on each branch of the phylogeny. Using this strategy, we were able to identify a number of expanding and contracting gene families in *P. brassicum* P1 (Table 3). Major expanding domain families included Ankyrin repeats, which play a role in protein–protein interaction; reverse transcriptase; a number of protein families involved in chromatin remodeling (e.g., SET domain proteins, chromatin organization modifier domain proteins, and centromere DNA binding proteins); and the integrase core domain, which is responsible for retroviral incorporation into the host genome. Major contracting families included a number of transporter or facilitator families, such as: ABC transporters, major facilitator superfamily, transmembrane amino acid transporters, and sugar transporters. The contractions seen in transporter families in *P. brassicum* P1 may be the result of lacking diversity in nutrient uptake and therefore host range.

### 2.5. Core and Species-Specific Gene Families

**Figure 3:**
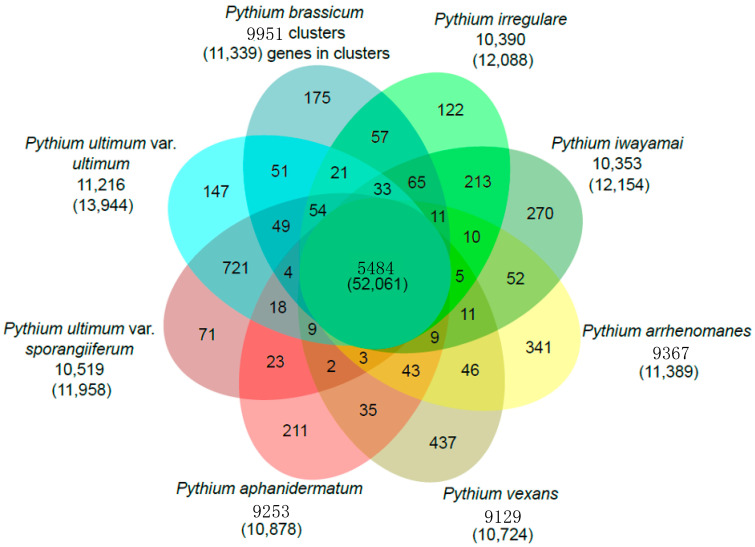
Venn diagram showing gene families shared by *P. brassicum* P1 and other *Pythium* species (i.e., *P. ultimum* var. *ultimum* and *P. aphanidermatum*).

We compared the genome content of *P. brassicum* P1 and seven other previously published *Pythium* genomes to identify species-specific gene clusters, as well as a core *Pythium* genome using OrthoMCL [25]. In general, *Pythium* species had genes contained in ~9000 to ~11,000 gene clusters. The *Pythium* core genome contains a total of 5484 orthologous gene clusters, made up of 52,061 total proteins across the genus. Figure 3 shows a comparison of all *Pythium* species analyzed and the number of gene clusters and genes shared between species. *Pythium brassicum* had 175 species-specific gene clusters, which was slightly below average for the species used in this comparison. Secondly, we performed targeted analysis of the identified *P. brassicum* P1 proteome, for example analysis of the secretome, effectors, proteins involved in carbohydrate metabolism, etc.

### 2.6. Secretome

Using SignalP [26], we identified secreted proteins in the *P. brassicum* P1 genome. There are 633 proteins (4.78% of the proteome) that are predicted to be secreted in the *P. brassicum* P1 genome, which is, again, slightly below average among published *Pythium* genomes (genomes downloaded from the *Pythium* genome database and annotated for secreted genes using the same method as *P. brassicum*). Notable protein families in the *P. brassicum* P1 secretome included aspartyl proteases, cysteine proteases, cytochrome p450s, elicithin-like proteins, glycoside hydrolases, lipases, NPP1-like proteins, carbohydrate esterases, polysaccharide lyases, phospholipases, and protease inhibitors. The presence of so many proteinases in the secretome was not unexpected, given *P. brassicum* P1’s role as a plant pathogen; many of these genes would be expected to play a role in this species’ interactions with its host plants.

### 2.7. Ca^2+^-Dependent Cadherins

Cadherins are calcium ion-dependent transmembrane proteins that are involved in the formation of adherens junctions responsible for binding cells together [27]. *Pythium ultimum* had four cadherin genes with calcium ion-binding LDRE and DxND motifs [19]. In contrast, *P. brassicum* P1 contained only one cadherin gene in its genome.

### 2.8. Effector Repertoire

**Figure 4:**
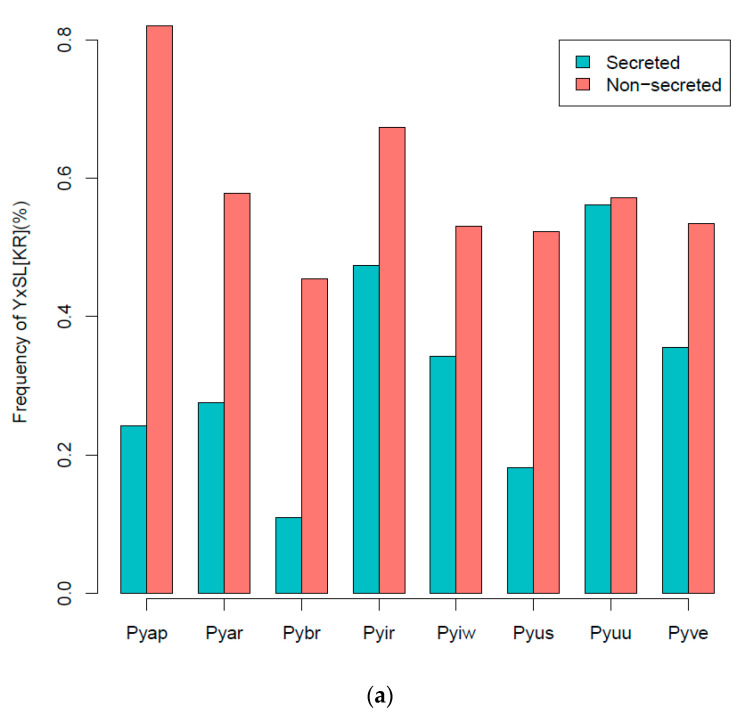

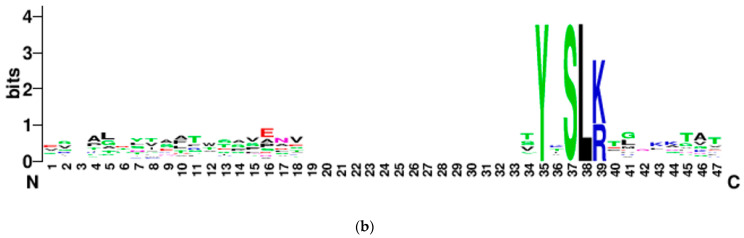
Percentage frequency of secreted and non-secreted YxSL[RK] effector proteins in *P. brassicum* P1 (Pybr), *P. aphanidermatum* (Pyap), *P. arrhenomanes* (Pyar), *P. irregulare* (Pyir), *P. iwayamai* (Pyiw), *P. ultimum* var. *sporangiiferum* (Pyus), *P. ultimum* var. *ultimum* (Pyuu), and *P. vexans* (Pyve) (**a**), and the typical architecture of a YxSL(RK) effector candidate inferred from 51 *Pythium* YxSL(RK) protein motifs (**b**). The consensus sequence pattern of YxSL(RK) motif was computed using WebLogo (available online: http://weblogo.berkeley.edu/logo.cgi) (accessed on 16 February 2021). The larger the letter, the more conserved the amino acid site. The numbers in the sequence logo refer to the corresponding positions in the alignment and thus differ from the average position of the motifs in the proteins.

**Figure 5:**
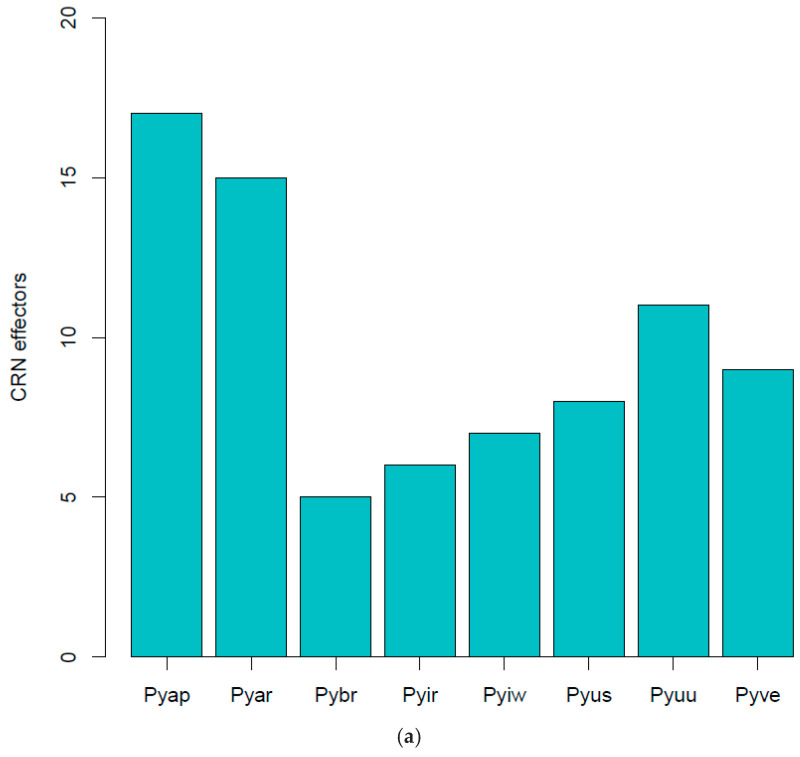

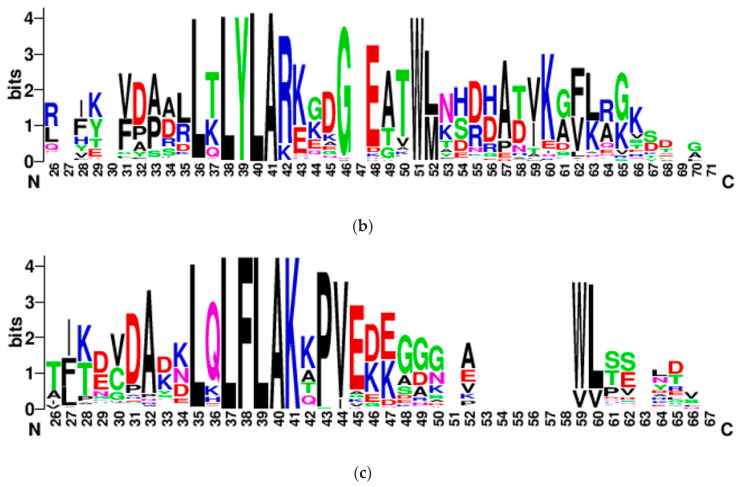
CRN effectors showing distribution in various *Pythium* spp. (**a**), abbreviations as in Figure 4, LYLAR or LYLAK motif (**b**), and LXLFLAK motif (**c**). The number of candidate CRN effectors in each genome was estimated as above with YxSL[RK] effectors.

Using the predicted secreted proteins and an HMM search, we identified candidate effector proteins in previously identified classes (YxSL, CRN, and RxLR):

(i). YxSL[KR] effectors: *P. brassicum* P1 had much smaller proportions of the YxSL sequence motif in both secreted and non-secreted proteins, relative to other *Pythium* species. *Pythium ultimum* var. *ultimum* had the highest proportion of secreted proteins with YxSL motifs, while *P. aphanidermatum* had the highest proportion of non-secreted proteins with YxSL motifs. *Pythium brassicum* P1 had the lowest proportion of proteins with YxSL motifs in both secreted and non-secreted proteins (Figure 4a,b).
(ii). CRN effectors: The *Crinkler* (*crn*) gene family encodes a large class of secreted proteins that share a conserved amino-terminal LFLAK domain involved in host translocation in *Phytophthora* spp. [23]. As seen with YxSL effectors, *Pythium brassicum* P1 had the fewest CRN effectors of all the *Pythium* species (Figure 5).
  - LYLAR or LYLAK motifs: *P. brassicum* P1 was predicted to have three secreted proteins with the LYLA[R/K] motif, which was below the *Pythium*-wide average of 11.75 (Figure 5a). The genome was predicted to have 109 non-secreted proteins with the LYLA[R/K] motif, again below the *Pythium*-wide average of 240.25 (Figure 5b).
  - LxLFLAK motif: We found no evidence for the LxFLAK motif in secreted proteins from any of the *Pythium* genomes, except for *Pythium arrhenomanes*, which had one (Figure 5c). There were similarly low numbers of non-secreted proteins in *Pythium* genomes with the LxLFLAK motif.

(iii). RxLR effectors: Consistent with previous studies, we found no evidence of RxLR virulent effectors in the *P. brassicum* P1 genome. This is in contrast to *Phytophthora* spp., which contain hundreds of RxLR genes in their genomes. These effector proteins are known to have an amino-terminal cell-entry domain with the RxLR and dEER motifs [23,28] that mediate the entry of these effector proteins into host cells without requiring the presence of pathogen-encoded machinery [29]. The RxLR-dEER effectors are thought to be involved in manipulating host immunity and suppressing host defense responses, but a few are recognized by plant immune receptors, culminating in programmed cell death and disease resistance.

The general reduction across all the effector classes in *P. brassicum* P1 is likely a result of the switch to host specialization in this species. As fewer hosts are utilized, a less diverse effector repertoire would be required to invade and colonize those hosts.

### 2.9. Carbohydrate Metabolism

We also annotated the carbohydrate-active enzymes in *Pythium* and other oomycete genomes using the CAZy database [30]. Carbohydrate-active enzymes aid in breaking down cell walls and other components of plant cells [31]. In general, *P. brassicum* P1 had an average number of proteins falling in the “Auxiliary Activities” category for *Pythium* species (*P. brassicum* P1: 20 genes in category, *Pythium* average: 20.75), a nearly average number of proteins in the “Carbohydrate Binding Module” category (P1: 50, *Pythium* average: 51.875), a below average number of carbohydrate esterases (P1: 43, *Pythium* average: 53.25), a below average number of glycoside hydrolases (P1: 133, *Pythium* average: 138), a slightly below average number of glycosyl transferases (P1: 104, *Pythium* average: 107.625), and a below average number of polysaccharide lyases (P1: 12, *Pythium* average: 16.125).

**Figure 6:**
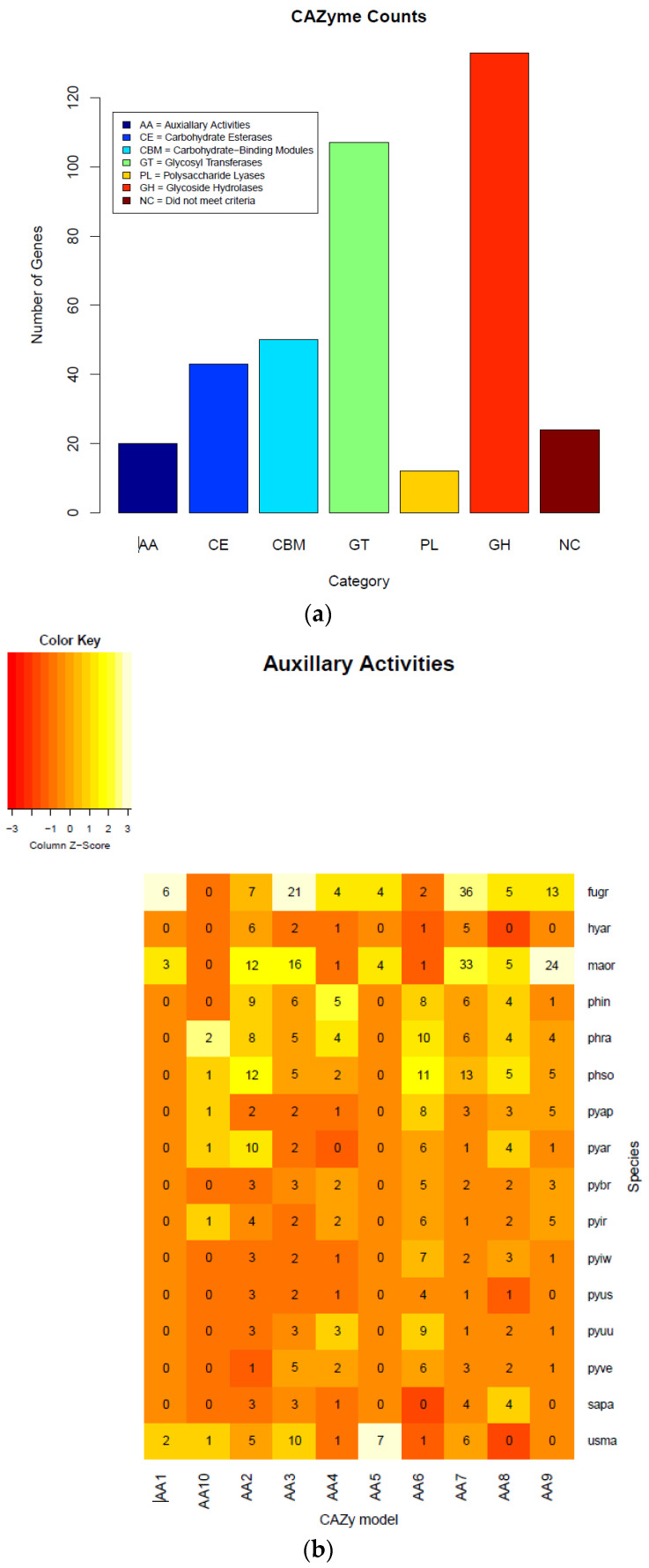

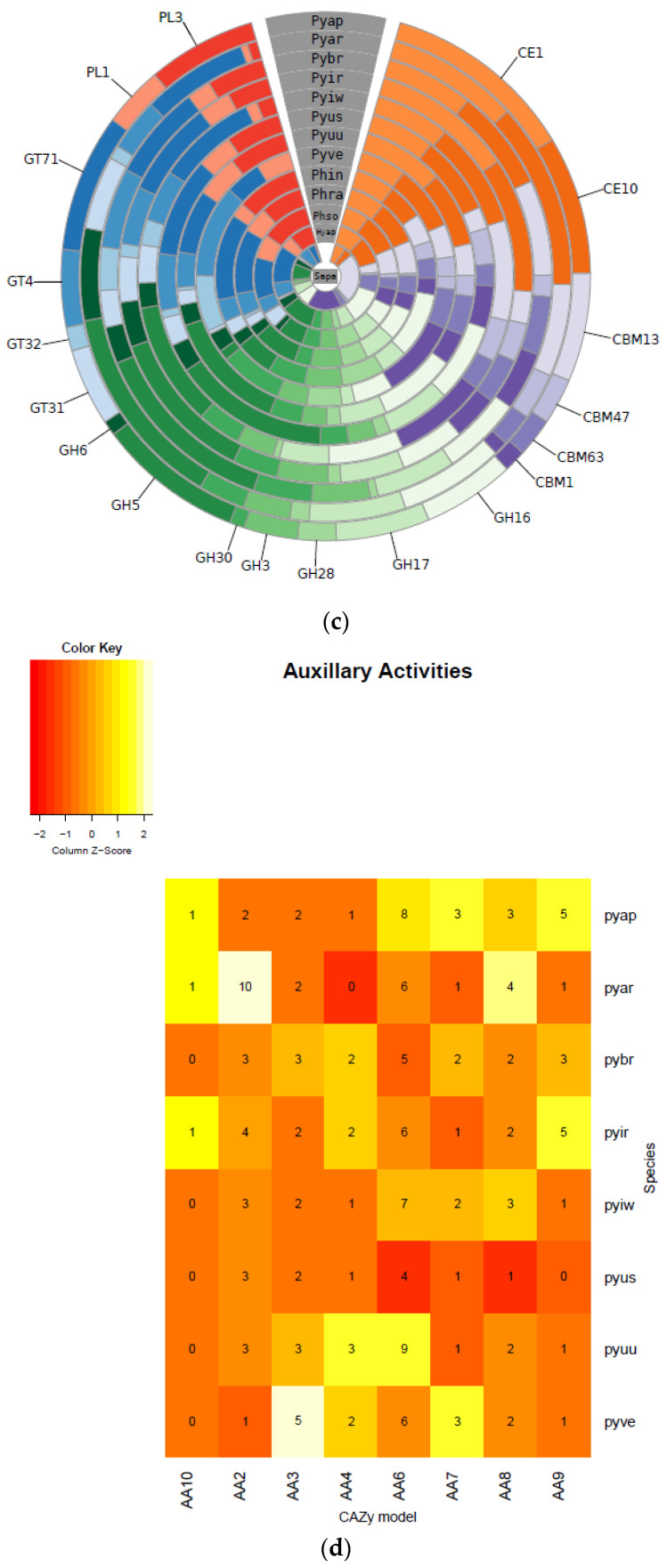
Carbohydrate-active enzymes (CAZymes) plots of each class (**a**), heatmaps (**b**), circos (**c**), and *Pythium* specific CAZy analysis (**d**). Annotation of the CAZyme-coding genes was done using the CAZymes Analysis Toolkit-CAT based on the CAZy database in combination with protein family domain analysis.

Among *Pythium* species, *P. brassicum* P1 had a reduced number of proteins falling under carbohydrate binding module (CBM) 47, which plays a role in fucose binding; glycoside hydrolase (GH) 12, a xyloglucan hydrolase; GH 81, an endo-β-1,3-glucanase; carbohydrate esterase (CE) 1, a family that contains acetyl xylan esterases, cinnamoyl esterases, and carboxylesterases, among others; and CE 10, a family that contains acetylcholinesterases, cholinesterases, and sterol esterases. *Pythium brassicum* P1 showed increased numbers of CE 4, a family that includes chitin deacetylases, chitooligosaccharide deacetylases, and peptidoglycan GlcNAc deacetylases; GH 7, a family that includes reducing end-acting cellobiohydrolases and chitosanases; glycosyl transferase (GT) 48, a 1,3-β-glucan synthase; and GT 32, which includes α-1,6-manosyltransferases and inositol-phosphorylceramide transferases (Figure 6a–d, Table 4).

The total number of candidate glycoside hydrolases (GHs) identified in *P. brassicum* P1 was 133. This is compared to 180 candidate GHs reported in *P. ultimum* [19]. Similar to *P. ultimum* [19], *P. brassicum* P1 did not possess any candidate cutinases in its genome, suggesting that, like *P. ultimum*, *P. brassicum* P1 infects host plants through non-suberized young roots as well as wounds. We did not identify any xylan degrading enzymes in the genome of *P. brassicum* P1, consistent with previous reports in *P. ultimum* and other *Pythium* spp. ([19] and references therein).

Pectin degrading enzymes or pectinases are known to play a key role in host plant infection by *Pythium* spp. *Pythium ultimum* is reported to have 29 candidate pectinase/pectin lyases [19] as compared to *P. brassicum* P1, which had only 12 predicted pectinase/pectin lyase. In addition to pectinases, *P. ultimum* has α-amylase, glucoamylase, and invertase genes that target starch and sucrose in the host plant [19]; three candidate starch and sucrose degrading enzymes were detected in the *P. brassicum* P1 genome. Again, the reduction in genes known to play a role in plant invasion in *P. brassicum* P1 is likely a result of the transition to host specificity.

### 2.10. Phylogenetic Position

**Figure 7:**
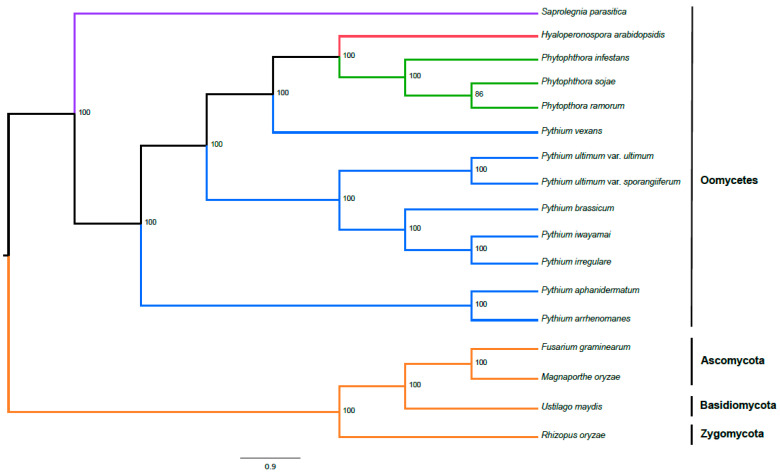
Phylogeny of *P. brassicum* P1 and other select oomycetes including *Saprolegnia*, *Hyaloperonospora*, *Phytophthora* and *Pythium* based on genome sequencing as inferred by maximum likelihood analysis. Outgroups include *Fusarium graminearum* and *Magnaporthe grisea* (Ascomycetes), *Ustilago maydis* (Basidiomycetes) and *Rhizopus oryzae* (Zygomycetes). Numbers on each node represent the percentage of bootstraps that support that node. Colors of the branches correspond to different genera (in the case of oomycetes) or outgroup fungi (orange: fungi; blue: *Pythium*; green: *Phytophthora*; red: *Hyaloperonospora*; and purple: *Saprolegnia*).

We used OrthoMCL [25] to identify single-copy orthologs across all published *Pythium* genomes, as well as several other oomycete and fungal genomes. We then aligned these single-copy orthologs and constructed a phylogenetic tree using RAxML [32] (Figure 7). *Pythium brassicum* P1 shared the most recent common ancestor with *P. iwayamai* and *P. irregulare*; that divergence was one of the more recent ones within *Pythium*, though there are three species pairs with more recent divergences. The next most recent common ancestor of *brassicum/iwayamai/irregulare* is shared with the two variants of *P. ultimum*. Together, these five species represent the only monophyletic *Pythium* clade in our tree. All other clades that included *Pythium* also included other oomycete species.

### 2.11. Shared Gene Clusters of Oomycetes

We further performed a comparison of important pathogenicity protein families among all oomycetes (Table 3). *Pythium brassicum* P1 showed a reduction in ABC transporters, aspartyl proteases, cytochrome p450s, and elicitin-like proteins. There were no important pathogenicity protein families in which *P. brassicum* P1 showed a large expansion. In general, *Pythium* species show reduced numbers of glycoside hydrolases, NPP1-like proteins, carbohydrate esterases, polysaccharide lyases, and protease inhibitors relative to *Phytophthora* species, and show no evidence of RxLR effectors. Again, there appears to be no important pathogenic proteins that show expansions in *Pythium* species relative to *Phytophthora* species. 

### 2.12. Orthologous Gene Clusters of Oomycete and Fungal Taxa

**Figure 8:**
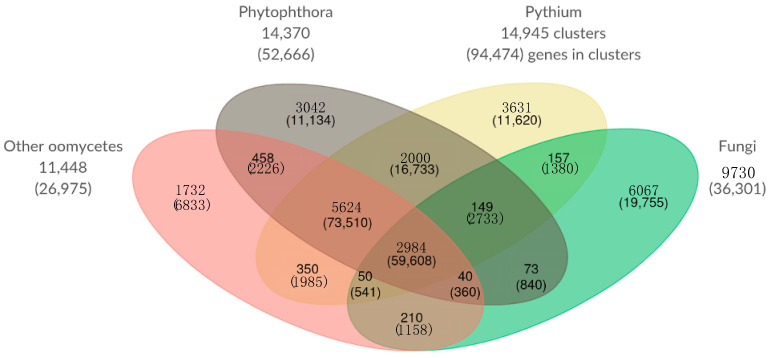
Comparison of orthologous gene clusters of *Pythium* species, *Phytophthora* species, Fungi, and other oomycetes. The number of gene clusters and total number of genes contained within those clusters (in parentheses) is displayed for each overlapping category. The numbers outside the Venn diagram show the total number of gene clusters (and genes) in each set.

Similar to our analysis of a *Pythium* core genome and species-specific clusters of orthologous genes above, we performed an analysis grouping our 8 *Pythium* genomes, 3 *Phytophthora* genomes, 2 other oomycete genomes, and 4 fungal genomes (Figure 8). In this analysis, *Pythium* species had 3631 unique clusters containing 11,620 genes; *Phytophthora* species had 3042 unique clusters containing 11,134 genes; the other oomycete species had 1732 unique clusters containing 6833 genes; and fungi had 6067 unique clusters containing 19,755 genes. There are 210 clusters and 1158 genes shared among all four classes analyzed.

### 2.13. Synteny with Other Oomycete Plant Pathogens

**Figure 9:**
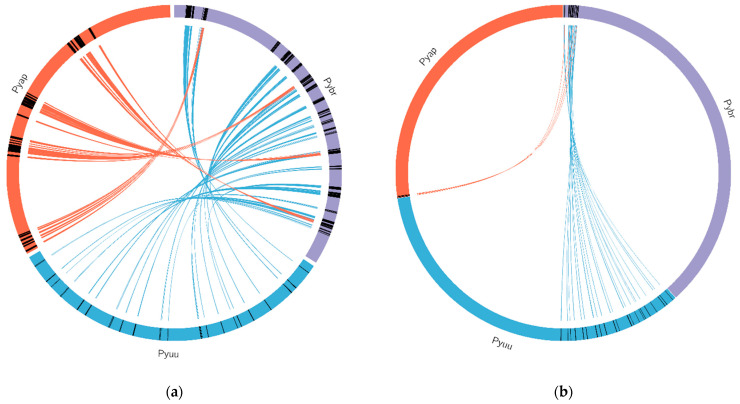

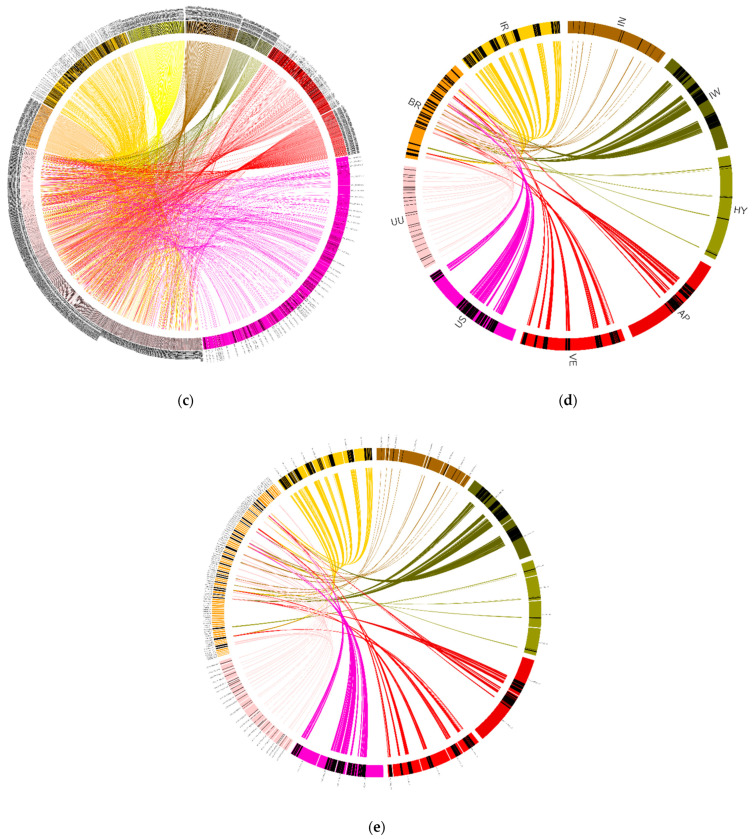
Synteny between the *Pythium brassicum* genome and several other oomycete genomes. Syntenic regions (as determined by MCScan) between *P. brassicum* and several other oomycete genomes is depicted. Lines connecting genomes indicate syntenic regions. (**a**) Synteny between select regions of *P. brassicum* (Pybr, purple), *P. ultimum* var. *ultimum* (Pyuu, blue), and *P. aphanidermatum* (Pyap, red). (**b**) Synteny across whole genomes of the species depicted in (**a**). (**c**) Synteny across selected genome regions for several oomycete species, scaled to the size of each genome (clockwise from top: *Ph. infestans*, *P. iwayamai*, *H. arabidopsidis*, *P. aphanidermatum*, *P. vexans*, *P. ultimum* var. *sporangiiferum*, *P. ultimum* var. *ultimum*, *P. brassicum*, *P. irregulare*, and *Ph. Ramorum*). (**d**) Synteny across select genome regions of several oomycete species, scaled so each genome is the same physical size on the graph (IN: *Ph. Infestans*, IW: *P. iwayamai*, HY: *H. arabidopsidis*, AP: *P. aphanidermatum*, VE: *P. vexans*, US: *P. ultimum* var. *sporangiiferum*, UU: *P. ultimum* var. *ultimum*, BR: *P. brassicum*, and IR: *P. irregulare*). (**e**) Same as (**d**) but scaled to the size of each genome.

A comprehensive analysis of synteny was carried out with all oomycete species using MCscan [33] (see Figure 9a–e). In general, we observed no evidence of large-scale inversions or rearrangements. We did, however, see some evidence of translocations in *Hyaloperonospora arabidopsidis* and *Pythium aphanidermatum*, relative to *P. brassicum* P1. Given that none of these genomes are resolved to chromosome level, these results must be met with caution.

## 3. Conclusions

*Pythium brassicum* P1 is an oomycete with a narrow host range infecting *mustard family* (*Brassicaceae*) only. This is in contrast to the majority of *Pythium* species, including *P. ultimum*, that have a wide host range infecting hundreds of diverse plant species. This study was thus designed to identify diverse biological parameters or mechanisms which might be responsible for P1’s narrow host range and where it could fit within a broader phylogenetic profile. We identified and sequenced the whole genome of a new *P. brassicum* P1 strain and compared to those with broad host range. Only a few species possess a narrow host range, and these include *P. iwayamai* and *P. arrhenomanes* which are pathogenic to monocotyledonous grasses. Both *P. ultimum* and *P. brassicum* P1 lack the hallmark RxLR effectors. One of the reasons for the absence of RxLR effectors in *Pythium* species is thought to be due to necrotrophic infection they cause on seedlings and stressed plants with weak defenses in contrast to other oomycete pathogens that possess RxLR effectors and are considered biotrophic, acquiring their nutrients from living cells. Most recently, Ai et al. [34] have reported the existence of functional RxLR effectors that induce tissue necrosis in several *Pythium* spp. including *P. utimum*. They argued that the existing genome annotation models seem to be inadequate for *RxLR* gene prediction and as a result they developed a modified regex model to allow the search for degenerate dEER motifs. *Pythium brassicum* P1 had three *Crinkler* (CRN) class of effectors with LYLA(R/K) motif compared to *P*. *ultimum* with 18 predicted CRN proteins [19], whereas *Phytophthora* spp. possess a large number of *Crinklers* that enter the host cells and trigger cell death and necrotrophy [23]. Like *P. ultimum* [19], *P. brassicum* P1 genome contained secreted proteins with a conserved RxLR-like motif (YxSL[KR]) that may act inside host cells during infection. Similar to *P. ultimum*, *P*. *brassicum* P1 lacked any cutinases suggesting that it may infect young seedlings through un-suberized root tissue as well as tissue wounds. This is in contrast to *P. arrhenomanes* and *P. aphenodermatum* that possess a total of 6 and 8 cutinase-encoding genes, respectively. The *P. brassicum* P1 genome encoded a much smaller number of cellulase and pectinase genes than *P. ultimum.* These genes facilitate initial penetration and infection of the host, and the narrower host range of *P. brassicum* P1 relative to *P. ultimum* may explain the reduction in the number of genes involved in host plant invasion. In vitro growth studies have shown that *P. ultimum* was unable to utilize complex polysaccharides such as xylan and chitin, but it easily degraded starch and sucrose [19,35]. Given that *P. brassicum* P1 similarly lacked xylanases, but had a limited set of pectinases, it would be expected that *P. brassicum* P1 possesses similar abilities to degrade starch and sucrose, though the range of these sugar molecules utilized by *P. brassicum* P1 may be limited. The inability of *P. brassicum* P1 to invade and colonize non-*Brassicaceae* species could be attributed, among other factors, to the lack of a wide repertoire of functional genes encoding cell wall degrading enzymes in its genome.

### Key Points

We identified and sequenced a new pathogen genome (named as *Pythium brassicum* P1) that infects only the *Brassicaceae* family of plants.

(i). Comprehensive bioinformatics analysis (e.g., comparison to 13 oomycete and 4 fungal outgroup species) revealed contracted regulation of metabolism, protein families, and distinct pathogenicity repertoire.
(ii). Assembled genome size is 50.3 Mb contained in 5434 scaffolds and 13,232 putative protein-coding genes identified; a detailed annotation analysis was performed.
(iii). Identified 175 species-specific gene families in *P. brassicum*, slightly below the normal average of other oomycetes, and a possible reason for the narrow host range of *P. brassicum.*
(iv). In contrast to other fungal or oomycetes, *P. brassicum* genome did not encode any classical RxLR effectors or cutinases, suggesting a significant difference in virulence mechanisms.
(v). A wide comparative analysis (e.g., over- and under-represented gene families, core specific gene families, secretome, Ca^2+−^ dependent adherens, effector repertoire, carbohydrate metabolism analysis, phylogenetic position, identification of shared and orthologous gene clusters, and synteny analysis with other plant pathogens) led to the identification of diverse biological parameters or mechanisms responsible for P1’s narrow host range.

## 4. Materials and Methods

### 4.1. DNA Extraction and Purification

*Pythium brassicum* isolate P1 was grown in 25 mL 10% (*v*/*v*) V8 juice broth, supplemented with 300 µg/mL vancomycin (to inhibit bacterial growth) at room temperature on a rotary shaker set at 150 rpm for seven days. V8 juice broth was inoculated with five agar plugs cut from the advancing mycelium of a three-day old V8 agar culture plate. The mycelia were vacuum-filtered on a Whatman filter paper placed on a Buchner funnel, washed a few times in sterile distilled water, blot-dried, and pulverized in frozen mortar and pestle using liquid nitrogen.

Genomic DNA was extracted using the protocol for yeast GenJET genomic DNA purification Kit (Thermo Fisher Scientific, Carlsbad, CA, USA). Briefly, 180 µL of digestion solution mixed with 20 µL protease K was added to the powdered mycelium in sterile centrifuge tube, mixed by vortexing and incubated at 56 °C for 45 min with occasional inversion. This was followed by adding 20 µL RNase A solution, mixing, and incubating at room temperature for 10 min. Two hundred µL lysis solution was added to the mixture, and the mixture was vortexed for 15 s. After adding 400 µL of 50% ethanol, the lysate was mixed and transferred onto GenJet column. The tube was centrifuged for at 8000× *g* for 1 min, flow through was discarded, column was placed on a new collection tube, 500 µL wash buffer I was added, tube was centrifuged for as above, flow through was discarded, column was washed with buffer II and centrifuged at 12,000× *g* for 3 min. Finally, 200 µL elution buffer was added to the column, incubated at room temperature for 2 min, and centrifuged for 1 min at 8000× *g*. Eluent containing DNA was run on agarose gel to examine for DNA integrity. DNA concentration and quality were measured using Nanodrop ND-1000 spectrophotometer.

### 4.2. DNA Library Preparation and Sequencing

Quality of genomic DNA template was analyzed by Agilent 2100 Bioanalyzer for Illumina sample preparation. For Next-Generation Sequencing, a total of 358 ng DNA in 130 µL was sheared using Covaris Focused-ultrasonicator™ Model S220 generating fragments with an average size of 436 bp. The NEBNext Ultra DNA Library Prep Kit for Illumina was used following the protocol provided with index#8 (New England BioLabs Inc., Ipswich, MA, USA).

The whole genome sequencing of *P. brassicum* P1 (CBS137315; MycoBank810861) was performed using Illumina HiSeq 2500. The run specifications were 2 × 101 × 7 cycles, version 3 flowcell, HCS 2.0.12.0, and RTA 1.17.21.3. The library was loaded at 10.0 pM across the flowcell which resulted in a cluster density of 747 k/mm^2^, a 91% Pass Filter rate, and 374 million total reads Passing Filter. The sequence Read 1 quality was 91.4% of bases ≥ Q30, and the sequence Read 2 quality was 86.9% of bases ≥ Q30.

### 4.3. Genome Assembly and Gene Prediction

Genome sequencing of the *P. brassicum* P1 was performed on a single library in a single lane of the Illumina HiSeq 2500 with 101 bp, paired-end reads. Barcode and adapter sequences were trimmed using the FASTX Toolkit (available online: http://hannonlab.cshl.edu/fastx_toolkit/index.html) (accessed on 26 February 2021), reads were filtered, and quality control was performed. Assembly was carried out on both the raw and filtered reads using Velvet [36], the String Graph Assembler (SGA) [37], and SOAPdenovo2 [17]. Velvet and SOAPdenovo2 assemblies were carried out with *k*-mers of 35–99, with a step size of four. SGA does not use a *k*-mer assembly, and the assembly was carried out with default parameters. Upon completion of assembly, the best assembly was selected (based on largest N50 and longest maximum scaffold length, and number of scaffolds) and used for further analysis. This assembly was then re-assembled with CAP3 program [18] using default parameters. The CAP3 reassembly program was repeat masked using RepeatScout software [22]. Gene prediction was carried out on the repeat masked assembly using the MAKER pipeline [20]. Seven previously published *Pythium* proteomes (downloaded from the *Pythium* Genome Database (http://pythium.plantbiology.msu.edu/, no longer available online)) were provided as evidence to the SNAP for gene model building and *P. ultimum* ESTs were provided to the MAKER to further refine the predictions. BUSCO (Benchmarking Universal Single-Copy Orthologs) was used to assess genome completeness [21]. The whole genome Shotgun project has been deposited in the NCBI/GenBank under the accession# ASM827159v1 (available online: https://www.ncbi.nlm.nih.gov/assembly/GCA_008271595.1/) (accessed on 15 August 2021).

### 4.4. Identification of Orthologous Groups

OrthoMCL [25] was used to identify clusters of orthologous genes among all of the genomes used in subsequent analyses. OrthoMCL started with an all-vs-all BLAST of all genes used in the analysis. These results were then filtered to remove hits of proteins to themselves, after which the Markov Cluster Algorithm, as implemented in MCL [38], was used to cluster proteins by similarity and orthologous clusters were constructed. The output from OrthoMCL was then used in a number of downstream analyses, outlined below.

### 4.5. Phylogenetic Analyses

A phylogeny of 13 oomycete species (8 Pythium, 3 Phytophthora, *Hyaloperonospora arabidopsidis*, and *Saprolegnia parasitica*) and four fungal outgroup species (*Magnaporthe oryzae*, *Fusarium graminearum*, *Rhizopus oryzae*, and *Ustilago maydis*) was constructed with RAxML [31]. Multiple sequence alignments of 341 single copy orthologs present in every genome, as determined by OrthoMCL [25], were aligned using MAFFT [39] and then passed to RAxML, which was run using the GAMMA model of rate heterogeneity and the LG model of substitution. One thousand bootstrap simulations were run, and the final tree was visualized using FigTree (available online: http://tree.bio.ed.ac.uk/software/figtree/) (accessed on 15 March 2021).

### 4.6. Analysis of P. brassicum P1 Over- and Under-Represented Families

Two methods were employed to determine the gene families that were significantly over- or under-represented in the *P. brassicum* P1 genome. The first was implemented in CAFE [40], which used a stochastic birth–death model to determine gene families that were significantly expanding or contracting (relative to ancestral state) along each branch of a phylogeny. Input for CAFE included the phylogenetic tree constructed with RAxML and the clusters of orthologous genes from OrthoMCL. After determining which gene families were significantly expanding or contracting on the branch leading to *P. brassicum* P1, a representative member from that family was selected and annotated with Pfam [41]. The second method used to determine over- and under-represented gene families in *P. brassicum* P1 was a one-to-one comparison of PANTHER protein family annotations [42] in the genomes of *P. brassicum* P1, and a generalist species of *Pythium*, *P. ultimum* var. *ultimum*. First, the set of PANTHER HMMs was downloaded from: http://data.pantherdb.org/ftp/panther_library/current_release/ (available online, accessed on 17 March 2021). Each of the two genomes in the analysis was then annotated for PANTHER protein family content using the script *pantherScore2.2.pl*, available here: http://data.pantherdb.org/ftp/hmm_scoring/current_release/pantherScore2.2/ (available online, accessed on 17 March 2021). After scoring each genome against the set of PANTHER HMMs, hits were filtered to include only those considered to be a close match, per the criteria laid out in the PANTHER manual. A list of *P. brassicum* P1 genes and their PANTHER annotations and *P. ultimum* var. *ultimum* genes and their PANTHER annotations were then uploaded to http://pantherdb.org/tools/compareToRefList.jsp (available online, accessed on: 15 March 2021), which used a Fisher’s exact test with false discovery rate correction to determine PANTHER families that were over-represented in one genome relative to another.

### 4.7. Identification of Putatively Secreted Proteins

The *P. brassicum* P1 predicted proteome was analyzed using the default parameters of SignalP [26] to identify proteins with secretion signals. Transmembrane domains were also predicted using TMHMM [43]. Proteins with: (i) no predicted transmembrane domains, (ii) SignalP Ymax score ≥ 0.5, (iii) SignalP D score ≥ 0.5, (iv) SignalP Smax score ≥ 0.9, and (v) SignalP secreted prediction equal to “Y” were considered as the secreted proteins of P1.

### 4.8. Analyses of Carbohydrate-Active Enzymes

All the genomes were further annotated for carbohydrate-active enzyme (CAZy) content [20] using the CAZymes Analysis Toolkit [44]. This method used two approaches to annotate the genome for CAZyme content: (1) a sequence similarity search against the entire CAZy database, and (2) an analysis of links between proteins and CAZymes using protein family domains.

### 4.9. Identification of Candidate Effectors

The known effector sequences for the effector classes that we looked at (YxSL, CRN, and RxLR) were downloaded from GenBank and aligned using MAFFT [39]. These alignments were used to create Hidden Markov Models for each effector class using HMMER (hmmer.org, version 3.1b2), after which the *hmmscan* algorithm in HMMER was used to search all protein sequences for all genomes used in our analyses against the profile HMMs created. Proteins that were identified as secreted as described above and that positively matched the profile HMMs were regarded as effectors falling into the respective class of the positive profile HMM. Further, string searches using Perl regular expressions were carried out to determine whether any potential effectors were missed using the methods above.

### 4.10. Synteny Analysis

All protein coding genes from all the 8 *Pythium* species used in the analyses in this paper were subjected to an all-vs-all BLASTP [45]. These results were used as the input for MCscan [33]. A python script contained in the MCscan package was used to filter the initial BLASTP results, remove self-hits, and order gene pairs for downstream analysis. Filtered BLASTP results were then clustered using the Markov Cluster Algorithm implemented in MCL [38]. The output of MCL, as well as the filtered/re-order BLASTP results and genomic BED files, were then supplied to MCscan to calculate pairwise synteny between *P. brassicum* P1 and all other *Pythium* genomes used in the analysis. The ‘-b’ option was used to limit within-genome synteny, all other parameters were left at program defaults. Custom Perl scripts were used to parse the MCscan output and generate input files for Circos [46], which was used to visualize the synteny among the genomes.

## Figures and Tables

**Table 1:** Number of contigs (**a**), cumulative length (**b**), and GC content (**c**) of *P. brassicum* P1 isolate in merged assembly, merged reassembly, sga processed data, sga raw data, soap processed data, soap raw data, and velvet processed data.

	Merged Assembly	Merged Reassembly	Sga_Processed Data	Sga_Raw Data	Soap_Processed Data	Soap_Raw Data	Velvet_Processed Data
# contigs (≥0 bp) ^a^	8759	9191	23,698	32,489	5437	5434	8420
# contigs (≥1000 bp)	4917	7631	5413	6977	3161	3074	6690
# contigs (≥5000 bp)	3454	4468	2336	36	2052	2020	3302
# contigs (≥10,000 bp)	2594	2661	1391	0	1468	1456	1569
# contigs (≥250,000 bp)	1161	852	420	0	624	631	207
# contigs (≥50,000 bp)	300	190	48	0	179	184	4
Total length (≥0 bp) ^b^	86,486,191	86,588,769	49,749,428	22,192,922	50,166,055	50,256,276	49,506,384
Total length (≥1000 bp)	85,411,003	85,824,021	44,743,758	12,211,453	49,404,430	49,485,932	48,588,674
Total length (≥5000 bp)	81,590,182	77,200,690	37,494,792	210,549	46,532,065	46,759,275	39,463,530
Total length (≥10,000 bp)	75,284,912	64,184,486	30,706,745	0	42,312,012	42,673,554	27,132,574
Total length (≥25,000 bp)	51,913,458	35,988,051	15,559,417	0	28,470,025	29,136,472	6,563,575
Total length (≥50,000 bp)	21,977,981	13,602,359	2,999,251	0	12,999,233	13,636,629	219,978
# contigs	5514	8341	7482	14,369	3629	3525	7543
Largest contig	169,369	168,160	108,107	8673	168,160	168,309	58,392
Total length	85,839,345	86,347,227	46,193,713	17,504,468	49,739,698	49,811,232	49,223,359
GC (%) ^c^	59.81	59.63	59.95	59.94	59.59	59.61	59.59
N50	31,156	20,060	16,383	1400	30,050	30,476	11,201
N75	16,898	9789	7080	907	15,561	15,907	5942
L50	841	1176	799	4050	493	478	1330
L75	1772	2720	1860	7938	1073	1044	2837
# N’s per 100 kb	1386.58	345.10	2036.02	4612.90	613.35	560.43	0.00
predicted genes (unique)	24,973	26,049	12,908	-	14,305	14,423	-
# predicted genes (≥0 bp)	88,235	90,798	59,287	-	52,918	51,696	-
# predicted genes (≥300 bp)	31,133	32,154	18,350	-	18,234	17,990	-
# predicted genes (≥1500 bp)	5790	5744	1714	-	3191	3321	-
# predicted genes (≥3000 bp)	1383	1271	209	-	703	789	-

All statistics are based on contigs of size ≥ 500 bp, unless otherwise noted (e.g., “# contigs (≥0 bp)” and “Total length (≥0 bp)” include all contigs).

**Table 2:** *Pythium brassicum* assembled genome statistics.

	Scaffolds	Contigs
Number of sequences	5434	64,712
Maximum sequence length (bp)	168,309	56,387
Average length (bp)	9248.49	879.7
N50(bp)	30,235	6705
N90 (bp)	6892	207
**Sequences > 500 bp**		
Number of sequences	3525	11,344
Average length (bp)	14,130.85	4279.21
N50(bp)	30,476	8290
N90 (bp)	7473	1811
**Sequences > 1 Kb**		
Number of sequences	3074	8364
Average length (bp)	16,098.22	5551.03
N50(bp)	30,985	8732
N90 (bp)	7803	2396
**Sequences > 5 Kb**		
Number of sequences	2020	3090
Average length (bp)	23,148.16	10,840.34
N50(bp)	33,000	12,061
N90 (bp)	10,751	6102
**Sequences > 10 Kb**		
Number of sequences	1456	1273
Average length (bp)	29,308.76	16,327.77
N50(bp)	35,695	16,489
N90 (bp)	14,907	11,104
Total number of assembled bases	50,256,276	

**Table 3:** The number of proteins in *Pythium brassicum* P1 genome with a single copy (‘Single hits’) or multiple copies (‘Multi hits’) of domains involved in host plant disease development.

Description	Multi_Hits	Single_Hits
ABC transporter transmembrane region	0	17
Transmembrane amino acid transporter protein	0	5
ABC transporter	0	5
Major facilitator superfamily	0	4
Sugar (and other) transporter	0	4
Sulfatase	0	2
Alcohol dehydrogenase GroES-like domain	0	2
Zinc Binding dehydrogenase	0	2
AMP-binding enzyme	0	1
Uncharacterized protein family UPF0565	0	1
RecF/RecN/SMC N terminal domain	0	1
HECT–domain (ubiquitin–transferase)	0	1
Putative transposase DNA-binding domain	0	1
Tc5 transposase DNAbinding domain	0	1
AAA domain, putative AbiEii Toxin, type IV TA System	0	1
Reverse transcriptase-like	0	1

**Table 4:** Number of proteins in protein families known to be involved in host plant disease development in *P. brassicum* P1 and other oomycetes and fungal species.

	Pap ^a^	Par	Pbr	Pir	Piw	Pus	Puu	Pve	Phi	Phr	Phs	Har	Sap	Fgr	Mor	Uma	Ror
ABC transporters	171	165	90	205	246	177	247	243	214	253	241	73	223	106	87	70	79
Aspartyl proteases	33	36	25	28	24	24	49	20	15	58	65	10	16	26	22	14	150
Cutinases	9	7	0	0	0	0	0	0	4	4	15	20	0	12	18	4	0
Cysteine proteases	29	35	37	36	39	32	37	28	33	35	33	22	79	6	7	5	1
Cytochrome P450s	31	60	12	53	66	32	39	27	26	29	38	14	44	110	134	22	48
Elicitin-like proteins	37	41	24	45	34	27	43	30	42	77	56	16	23	0	0	0	0
Glycoside hydrolases	117	163	133	133	118	110	168	162	273	271	294	98	198	259	266	125	U ^b^
Lipases	26	26	22	15	11	10	21	24	31	26	47	11	49	40	30	11	37
NPP1-like proteins	4	5	3	4	4	4	7	4	28	58	80	32	0	4	5	0	0
Carbohydrate esterases	68	75	43	56	41	29	63	51	76	92	129	34	73	130	125	61	U
Polysaccharide lyases	22	7	12	14	7	16	29	22	66	53	76	15	5	21	5	2	U
Phospholipases	20	23	15	16	15	11	18	19	31	28	31	16	18	41	26	14	17
Protease inhibitors	27	23	21	28	19	22	31	14	60	25	59	2	14	0	0	0	0
RxLR effectors	0	0	0	0	0	0	0	0	563	350	350	7	0	0	0	0	0

^a^ Pap, Pythium aphanidermatum; Par, Pythium arrhenomanes; Pbr, Pythium brassicum; Pir, Pythium irregulare; Piw, Pythium iwayamai; Pus, Pythium ultimum var. sporangiiferum; Puu, Pythium ultimum var. ultimum; Pve, Pythium vexans; Phi, Pythophthora infestans; Phr, Phytophthora ramorum; Phs, Phytophthora sojae; Har, Hyaloperonospora arabidopsidis; Sap, Saprolegnia parasitica; Fgr, Fusarium graminearum; Mor, Magnaporthe oryzae; Uma, Ustilago maydis; Ror, Rhizopus oryzae. ^b^ Undetermined.

## Data Availability

The whole genome shotgun project data have been deposited in the NCBI/GenBank and are available as accession # ASM827159v1 (available online: https://www.ncbi.nlm.nih.gov/assembly/GCA_008271595.1/) (accessed on 15 August 2021).

## Supplementary Materials

The following are available online at https://www.mdpi.com/article/10.3390/ijms22169002/s1.
