# Supplementary figures and images for: Insights into the Host Specificity of a New Oomycete Root Pathogen, Pythium brassicum P1: Whole Genome Sequencing and Comparative Analysis Reveals Contracted Regulation of Metabolism, Protein Families, and Distinct Pathogenicity Repertoire
Source: Int J Mol Sci. 2021 Aug 20;22(16):9002. doi: 10.3390/ijms22169002 (PMC8396444; doi:10.3390/ijms22169002)

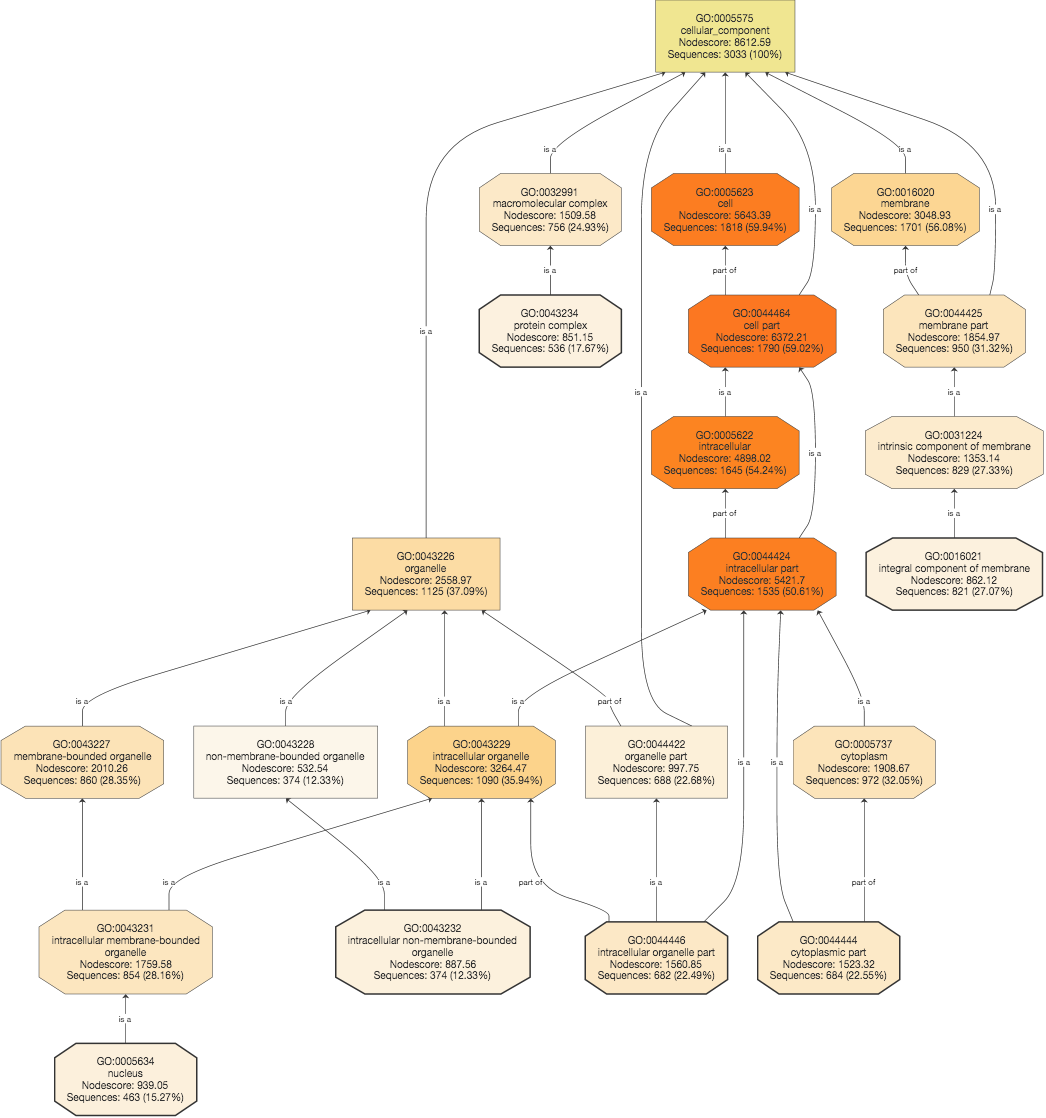

Supplement: Supplementary file 1 [file ijms-22-09002-s001.zip › Supplementary/S5-cellular_component_chart.png]

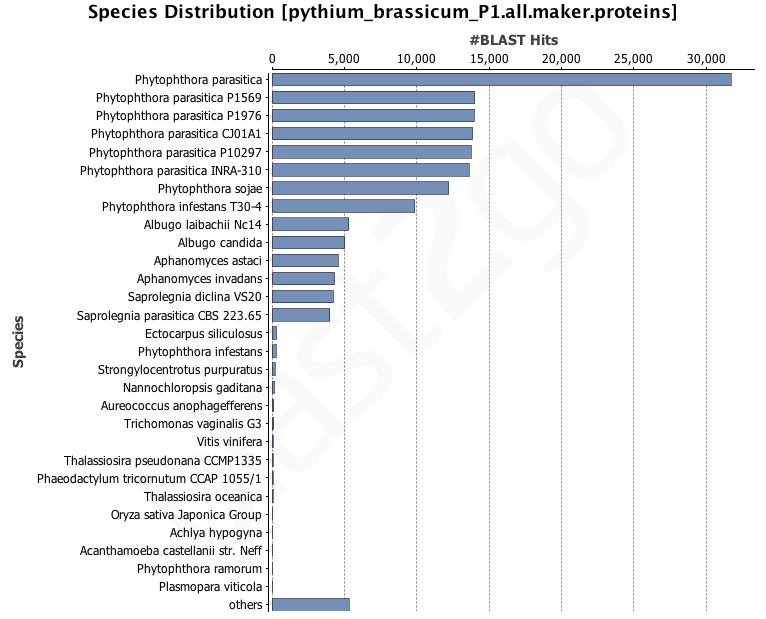

Supplement: Supplementary file 1 [file ijms-22-09002-s001.zip › Supplementary/S1-all_species_hits_distribution.png]

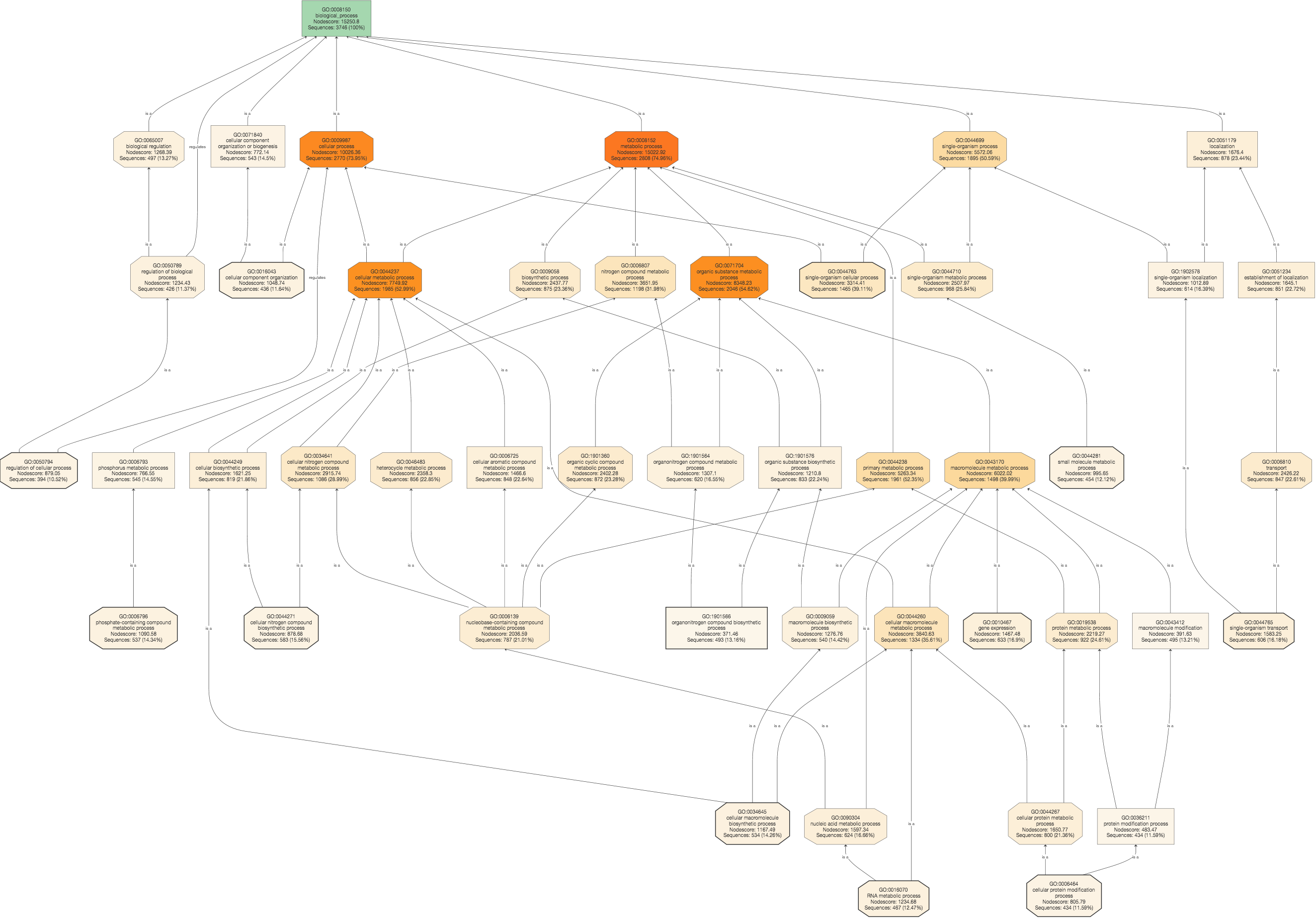

Supplement: Supplementary file 1 [file ijms-22-09002-s001.zip › Supplementary/S3-biological_process_chart.png]

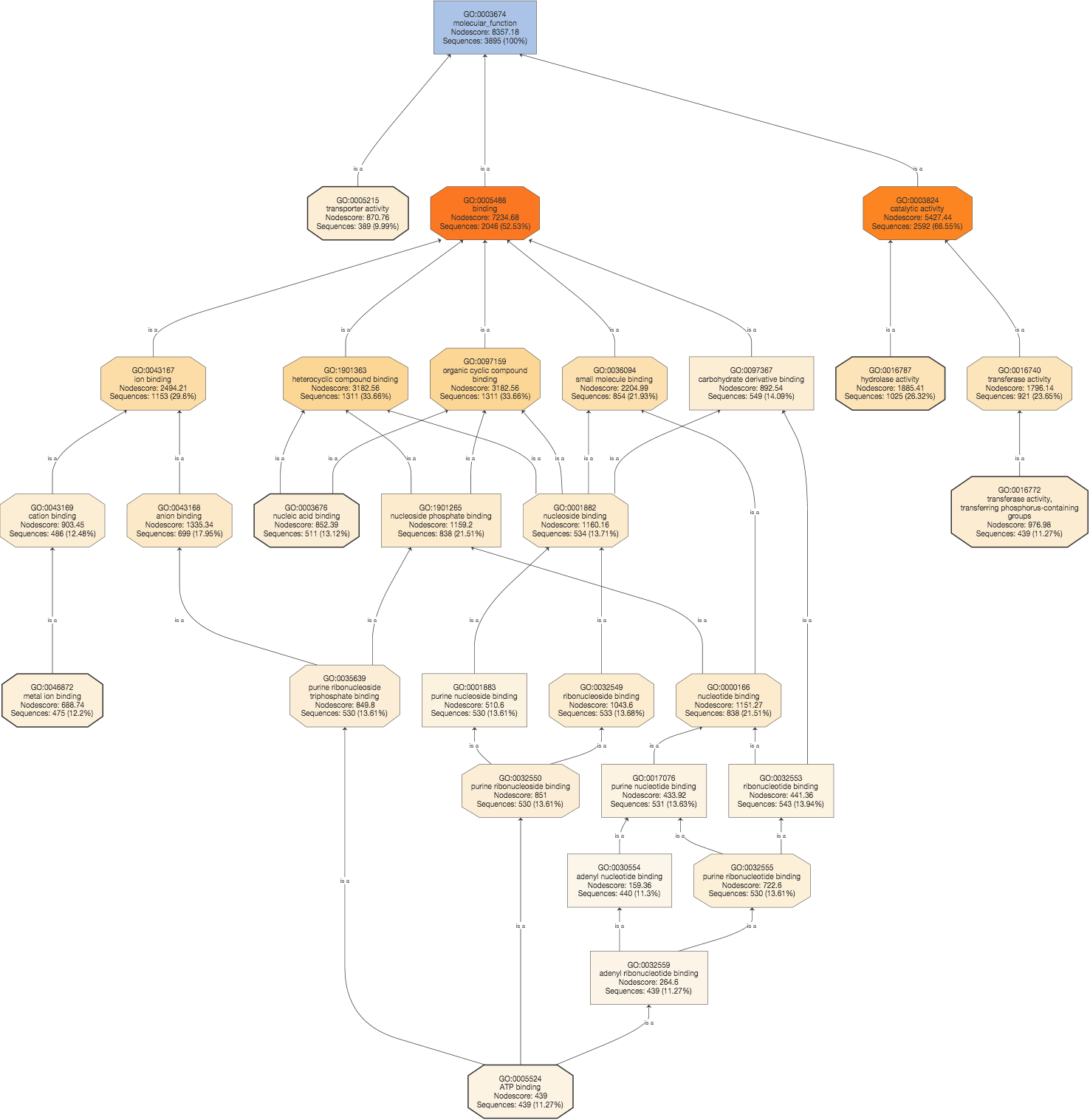

Supplement: Supplementary file 1 [file ijms-22-09002-s001.zip › Supplementary/S7-molecular_function_chart.png]

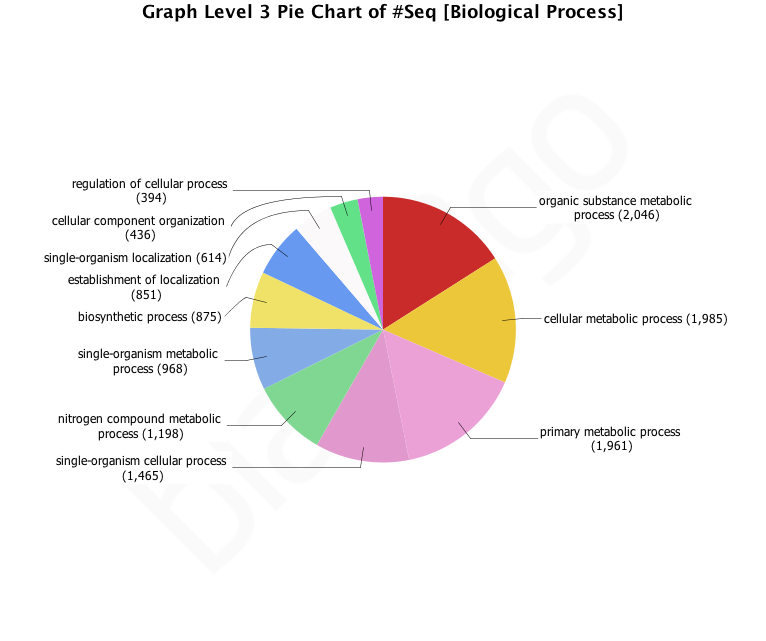

Supplement: Supplementary file 1 [file ijms-22-09002-s001.zip › Supplementary/S2-biological_process_level_3.png]

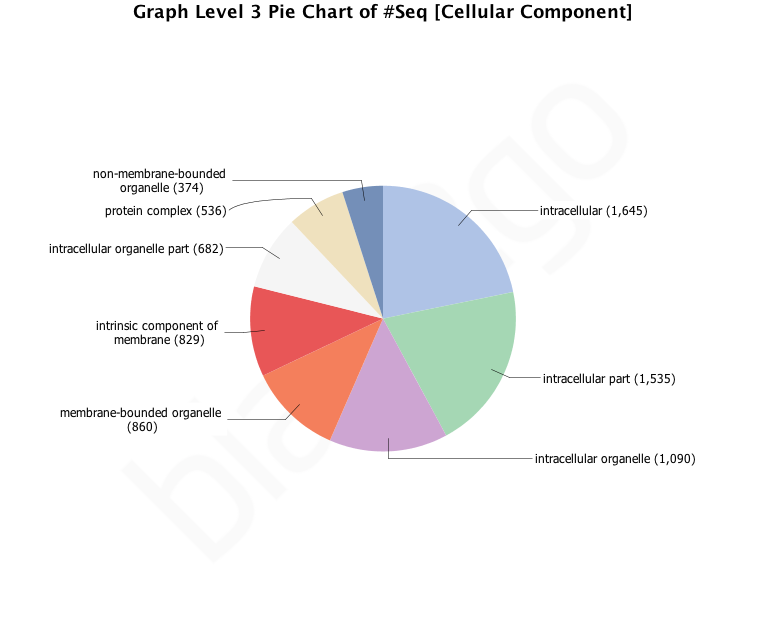

Supplement: Supplementary file 1 [file ijms-22-09002-s001.zip › Supplementary/S4-cellular_component_level_3.png]

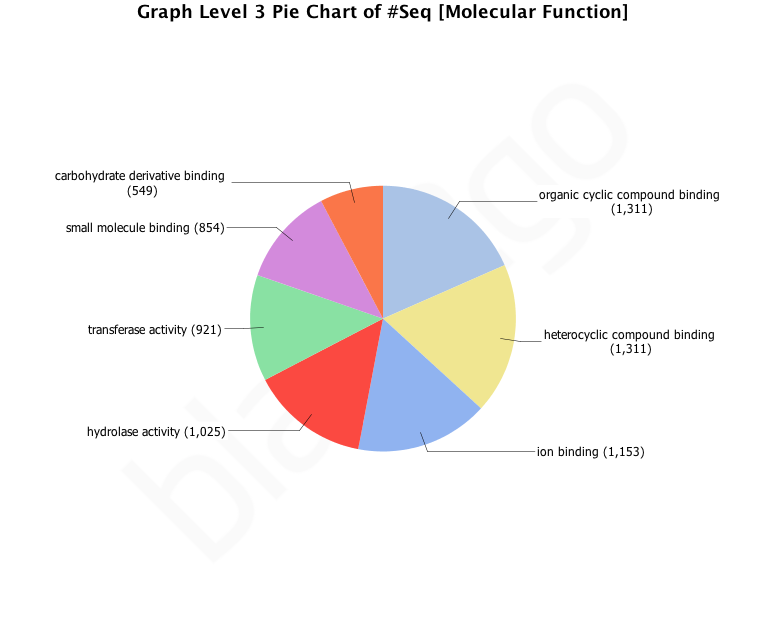

Supplement: Supplementary file 1 [file ijms-22-09002-s001.zip › Supplementary/S6-molecular_function_level_3.png]
